# Supplementary material for: Models of COVID-19 vaccine prioritisation: a systematic literature search and narrative review
Source: BMC Med. 2021 Dec 1;19:318. doi: 10.1186/s12916-021-02190-3 (PMC8632563; doi:10.1186/s12916-021-02190-3)
Supplement: Supplementary file 1 — Additional file 1. Models of COVID-19 vaccine prioritisation: a systematic literature search and narrative review includes Tables S1 to S9. Table S1 – Search strategy. Table S2 – Conclusions of studies on optimal vaccination priority group to minimise deaths from COVID-19 with study comparators. Table S3 - Exceptions to the majority of study conclusions on optimal vaccination priority group to minimise deaths from COVID-19. Table S4 - Conclusions of studies on optimal vaccination priority group to minimise cases of COVID-19 with study comparators. Table S5 - Exceptions to the majority of study conclusions on optimal vaccination priority group to minimise cases from COVID-19. Table S6 - Characteristics of the included UMIC and LMIC studies including supply and coverage assumptions made. Table S7 - Summary of the results of the sensitivity analyses conducted by the included studies and whether this influenced the recommendation of prioritisation strategy. Table S8 - Exceptions to the majority of study conclusions on optimal vaccination priority group to minimise deaths and cases of COVID-19 organised by level of coverage by the end of the modelled vaccination campaign. Table S9 - Summary of evidence from the systematic literature review supporting the prioritisation of groups in the WHO SAGE vaccine prioritisation roadmap under different supply scenarios during community transmission. [file 12916_2021_2190_MOESM1_ESM.docx]

**Additional file 1**

**Models of COVID-19 vaccine prioritisation: a systematic literature search and narrative review**

Authors: Nuru Saadi MSc ^1^*, Y-Ling Chi DPhil ^2^, Srobana Ghosh MSc ^2^, Rosalind M Eggo PhD ^3^, Ciara V. McCarthy MSc ^3^, Matthew Quaife PhD ^3^, Jeanette Dawa PhD ^4,5^, Mark Jit PhD ^3+^, Anna Vassall PhD ^1+^

*Correspondence: [Nuru.Saadi@lshtm.ac.uk](mailto:Nuru.Saadi@lshtm.ac.uk) and [nurusaadi@gmail.com](mailto:nurusaadi@gmail.com)

+ Joint senior authors

1. Department of Global Health and Development, London School of Hygiene and Tropical Medicine, London, UK

3. Centre for Mathematical Modelling of Infectious Diseases, London School of Hygiene and Tropical Medicine, London, UK

**This file includes:** Tables S1-S9

Table S1 – Search strategy for the systematic literature review

| Database | Search terms | Dates |
| --- | --- | --- |
| National Institute of Health iSearch COVID-19 portfolio  (peer-reviewed articles from Pubmed and Preprints from arXiv, bioRxiv, ChemRxiv, medRxiv, Preprints.org, Qeios, Research Square, and SSRN) | [title and abstract]​  ​  (1) model* AND vacc* AND (math* OR computation* OR simulat* OR optim*)​  (2) vaccin* AND (econom* OR cost) | 03/03/2021 |
| National Institute of Health iSearch COVID-19 portfolio  *Updated search* | [title and abstract]​  ​  model AND vaccination AND (mathematical OR computation OR simulation OR optimization)  Only Low and Middle Income country studies included during screening | 03/03/2021-24/09/21 |
| CEPR & NBER | All economics papers on COVID (hand search) | 03/03/2021​ |
| Econlit | [Title and abstract]​  Cost AND (covid-19 or coronavirus or 2019-ncov or sars-cov-2 or cov-19) | Entire database searched on 03/03/2021​ (filtered 2020-2021) |
| Econlit  *Updated search* | [Title and abstract]​  Cost AND (covid-19 or coronavirus or 2019-ncov or sars-cov-2 or cov-19)  Only Low and Middle Income country studies included during screening | Entire database searched on 29/09/2021​ (filtered 2020-2021) |

Table S2 – Conclusions of studies on optimal vaccination priority group to minimise deaths from COVID-19 with study comparators (n=34)

|  | **Study comparators** | | | | | | | | | | | |
| --- | --- | --- | --- | --- | --- | --- | --- | --- | --- | --- | --- | --- |
| **Conclusion on prioritisation to minimise deaths​** | **No group prioritisation​**  **​** | **No vaccination​** | **Adolescents/children​** | **Young adults​** | **Middle aged adults​** | **Seniors​** | **Comorbidities​** | **According to size of populations (sub & national)​** | **Social or work-related interaction​** | **Forecasting of infected individuals​** | **Other​^3^** |  |
| Seniors (26)^[[1]](#footnote-1)^ | [14], [15]​, [19] [20], [21], [22], [24], [32] | [14], [15], [17]​, [22], [23], [24], [26], [29] | [7], [11], [12],[28], [13], [16]​, [18]​, [19], [27], [31] | [7], [9], [10], [11], [12], [13], [15], [16], [17]​, [18]​, [20],[28], [23], [25], [27], [29], [30], [31],[32] | [8], [9], [10], [11], [12], [13], [15], [16], [17]​, [18]​,​ [19], [25],[28], [29], [30],[31] | [11]​, [18]​, [19], [26],[30] | [11], [15]​, [22], [26] | ​ [18] | [7]​, [11], [14] |  | [31] |  |
| Young adults (2) |  |  |  | [30]* | [30]* | [30]*,[39] |  |  |  |  |  |  |
| High social contact groups (4) | [35], [37], [38] |  | [29]*, [37],[38] | [29]*, [38] | [29]*, [37],[38] | [37],[38] |  |  | [37],[38] | [35],[38] | [35] |  |
| Essential workers (2) |  |  | [33] | [33] |  | [33], [34] |  |  |  |  | [34] |  |
| Other (4)^[[2]](#footnote-2)^ | [21] |  |  | [40] | [40] | [40] | [36] |  | [36] |  | [41] |  |

Table S3 - Exceptions to the majority of study conclusions on optimal vaccination priority group to minimise deaths from COVID-19

| Author​ | Conclusions​ |
| --- | --- |
| Luangasanatip et al​ | Prioritise young adults ​ |
| Chen (J) et al​ | Prioritise high social contact group ​ |
| Bonsall et al ​ | Prioritise the non-vulnerable group​ |
| Goldenbogen et al​ | Prioritise high social contact group​ |
| Moret et al​ | Prioritise high social contact group + seniors​ |
| Visscher et al​ | Prioritise children​ |
| Santini​ | Prioritise both seniors and the middle-age and young adults who have high-contact with them ​ |
| Rodriguez et al​ | Prioritise high social contact group​ |
| Buckner et al​ | Prioritise young adult and middle-age essential workers, then elderly​ |
| Babus et al | Prioritise middle-aged adult workers |

Table S4 - Conclusions of studies on optimal vaccination priority group to minimise cases of COVID-19 with study comparators (n=27)

| **Conclusion on optimal prioritisation to minimise cases** | **No group prioritisation​**  **​** | **No vaccination​** | **Adolescents/children​** | **Young adults​** | **Middle aged adults​** | **Seniors​** | **Comorbidities​** | **According to size of populations (sub & national)​** | **Allocation by geographic disease burden​** | **Social or work-related interaction​** | **Other​^6^** |
| --- | --- | --- | --- | --- | --- | --- | --- | --- | --- | --- | --- |
| **Young adults (13)^4^** | [12], [15]​, [20],[32] | [15], [17],[23] | [16], [12],[28],[31] | [16] [15]​,[31] | [16], [15]​, [27]​​,[28],[25],[31], [34] | [10], [16], [12], [15], [17]​, [20],[28],[23],[25],[27],[32],[34], [39] | [15] |  |  | [32],[34] | [31],[34] |
| **High social contact group (4)** | [37],[38],[43] | ​ | [37],[38] | [38] | [37],[38] | [37],[38],[43] | ​ | ​ | ​ | [37],[38], [42] | [43],[38] |
| **Essential workers (3)** | [14] | [14] | [33] | [33] | [34]* | [14]​, [33],[34]* |  |  |  | [14]​, [33],[34]* | [34]* |
| **Seniors (3)** |  |  | [13]​, [18]​,[31]* | [13]​, [18]​,[31]* | [13]​, [18]​,[31]* | [18] |  | [18] | [18] |  | [31]* |
| **Other (3)^5^** |  | ​[44] | ​​[44] | ​​[44] | ​[44] | ​[44] |  | ​​[45] | ​​[45],[46] | ​ | [40]​,[41],​[44],​[45] |

*Study appears twice

^4^ Also includes young and middle-aged adults, young adults and children

^5^ Other row: [41] & ​[44]by serology testing, ​[45] area of low disease burden, [46] area of high disease burden

Table S5 – Exceptions to the majority of study conclusions on optimal vaccination priority group to minimise cases from COVID-19

| Author​ | Conclusion​s |
| --- | --- |
| Chhetri et al​ | Prioritise seniors​ |
| Bertsimas et al​ | Prioritise geographic areas with higher projected cases and the seniors within those areas ​ |
| Hunziker et al​ | Prioritise seniors at full dose and young adults at quarter-dose in parallel ​ |

Table S6 - Characteristics of the included UMIC and LMIC studies including supply and coverage assumptions made

| F Author​ | Setting​ | Conclusion deaths​ | Conclusion cases​ | Supply and coverage​ |
| --- | --- | --- | --- | --- |
| Minoza​ | Philippines​ | Seniors​ | Mobile workforce (non-medical)​ | 500k vx to cover 1/6 of pop (unclear timing)​ |
| Foy​ | India​ | Seniors​ | Young and middle-aged adults​ | 6 months-4 yrs to cover 100% of target pop​ |
| Pearson​ | Pakistan​ | Seniors​ | ​ | 20% of pop phase 1. 4000 vx pd initially then increase in quarters over 1 year (base)​ |
| Meehan​ | 179 countries (detailed results for India, China & UK)​ | Seniors ​ | ​ | Depends on country and scenarios: up to 100% (timing not specified)​ |
| Bubar​ | Belgium; US; India; Spain; Brazil; Zimbabwe; South Africa; China ​ | Seniors​ | Young and middle-aged adults​ | 1-50% total pop supply (0.2% pd until supply exhausted)​ |
| Hogan​ | Global (HIC/UMIC/LMIC/LIC)​ | Seniors​ | ​ | Individuals vaccinated at a constant rate over a 1 month in country. 2 billion vx global constraint ​ |
| Luangasanatip ​ | Thailand​ | Young adults​ | Young adults​ | 9M vx in 2 months​ |
| Moret​ | Brazil and Portugal​ | High social contact group + seniors​  ​ | ​ | 250M vx for Brazil & 20M for Portugal available in one year​ |
| Gozzi | Egypt; Peru; Serbia; Ukraine; Canada; Italy | Seniors | Young adults in S, U & C. Uniform strategy in E & P | Daily vx 0.1%, 0.5% or 1.0% of the population. 100%, 80% or 60% coverage |
| España | Colombia | Seniors |  | 50,000/day to 100,000/day. 50-60% coverage |
| Mandal | India | Seniors |  | Unclear |
| Campos | Brazil | Seniors | Young adults | Vx 100, 150 or 200 days after the pandemic starts. Coverage not specified |
| Fuady | Indonesia |  | Districts with highest incidence | 464k - 232k vaccines.  33,5 million people within six months |
| Goldstein | United States; Japan;  Bangladesh | Seniors |  | A constant share of the population each day until 100% of the pop has been vaccinated |
| Yu | China | Seniors | Young adults | 2.0 million courses supplied per day (0.14% rollout speed). Coverage depends on the scenario and age group |

Table S7 - Summary of the results of the sensitivity analyses conducted by the included studies and whether this influenced the recommendation of prioritisation strategy

| **Studies** | **Outcomes** | **Original priority** | **Priority changed in sensitivity analysis** | **Sensitivity analysis that changes the priority** |
| --- | --- | --- | --- | --- |
| Ayoub | Cases | Serology based | No |  |
| Babus, A | Cases | Middle aged adult workers | Yes | Increasing vaccine supply would decrease the age of the youngest eligible recipients. |
|  | Deaths |  |  |  |
| Bertsimas, D | Cases | E/V | No |  |
|  | Deaths | E/V |  |  |
| Bonsall, M | Deaths | Y/HC | No |  |
| Brüningk, S | Cases | HC (low SES) | No |  |
| Bubar, K | Cases | Y/HC | No |  |
|  | Deaths | E/V | Yes | Change priority to 20-49 if:  VE low in older adults, R0 = 1.15 and VE >80% transmission blocking, R0=1.15 and rollout speed 0.2% per day or vaccine for 25% of the population, leaky vaccines (prioritise children). |
| Buckner, J | Cases | Y/HC | No |  |
|  | Deaths | Y/HC |  |  |
| Campos, E | Cases | Y/HC | Yes | For lower transmission rates it is always better to vaccinate the elders. For higher transmission rates, these results are reversed  Optimal policies depend, among other things, on transmission rates, the vaccination efficiency and its timing. Earlier vaccination dates, low vaccine efficiency and higher transmission indices increase the impact of vaccinating the young. |
|  | Deaths | E/V |  |  |
| Castonguay, F | Cases | Area with the lower disease burden | No |  |
| Chen, J | Cases | Y/HC | No |  |
|  | Deaths | Y/HC |  |  |
| Chen, X | Cases | E/V | Yes | For dynamic policies, the older groups should be vaccinated at early days and then switch to younger. |
|  | Deaths | E/V |  |  |
| Chhetri, B | Cases | E/V | No |  |
|  | Deaths | E/V |  |  |
| Espana, G | Deaths | E/V | Yes | If delta dominance occurred after mid September, age prioritization would be less relevant |
| Faudy, A | Cases | Area with the higher disease burden | No |  |
| Foy, B | Deaths | E/V | No |  |
|  | Cases | Y/HC |  |  |
| Fujimoto, A | Cases | Serology based | No |  |
|  | Deaths |  |  |  |
| Goldenbogen, B | Cases | Y/HC | Yes | For high vaccination levels the strategy to vaccinate the most interactive individuals first is most effective for all three objectives. There is a trade-off between different strategies for low levels of vaccination. |
|  | Deaths | Y/HC |  |  |
| Goldstein, J | Deaths | E/V | No |  |
| Gozzi, N | Cases | E/V | No |  |
|  | Deaths | Y/HC, all |  |  |
| Grundel, S |  | Y/HC | No |  |
| Guerstein, S |  | E/V | No |  |
| Hogan, A | Deaths | E/V | Yes | For limited supply (<20%) target elderly/vulnerable. For supply >20% switch to targeting key transmitters (potentially include children) for indirect protection. |
| Hoertel, N | Cases | Y/HC | Yes | With lower supply prioritise the older population. |
|  | Deaths | E/V |  |  |
| Hunziker, P | Cases  Deaths | Prioritise seniors at 100% and younger at 25% dose |  |  |
| Jentsch, P | Deaths | E/V | Yes | If seropositivity is high (particularly if roll out is delayed) or VE is lower in vulnerable groups, prioritise to interrupt transmission. |
| Kirwin, E | Cases | Equal priority to all groups | Yes | Rank ordering of different prioritisation options varied greatly by prioritisation criteria, with different vaccine effectiveness and coverage, and by concurrently implemented policies. |
| Luangasanatip, N | Cases | Y/HC | Yes | VE reduction in severity 70-90% and reduced susceptibility 0%, prioritise vulnerable. |
|  | Deaths | Y/HC |  |  |
| Macintyre, C R | Cases | Y/HC | No |  |
|  | Deaths | E/V |  |  |
| Mandal, S | Deaths | E/V | Yes | Direct effects of immunisation take precedence in deciding prioritisation when the vaccine supply is sufficient to cover 18% of the population. In the lowest transmission settings those with comorbidities would be prioritised over the elderly. |
| Matrajit, L | Cases | Y/HC | Yes | Vaccine coverage for approx. half the population with VE>60%, prioritise high transmission. If more vaccine becomes available, prioritise the vulnerable again. (Depends on pre-existing immunity). |
|  | Deaths | E/V |  |  |
| Meehan, M | Cases | Y/HC | No |  |
|  | Deaths | E/V |  |  |
| Minoza, J | Cases | Mobile work force | No |  |
|  | Deaths | E/V |  |  |
| Moore, S | Deaths | E/V | Yes | When R>1 and there is a race to herd-immunity to prevent further rise a rapid vaccine deployed untargeted campaign is far more successful than a slow but optimally targeted one. If vaccine efficacy is significantly reduced in the elderly (<20%) then other orderings become more effective. |
| Moret, M | Deaths | E/HC | No |  |
| Pearson, C | Deaths | E/V | No |  |
| Rodriguez, J | Deaths | HC | No |  |
| Shayak, B | Cases | Y/HC | No |  |
| Shim, E | Cases | Y/HC | Yes | Supply >80% incidence-minimizing strategies lead to a broader vaccination strategy for those aged 10–69 years. |
|  | Deaths | E/V |  |  |
| Tran, T | Cases | Y/HC | No |  |
|  | Deaths | E/V |  |  |
| Visscher, A | Deaths | Children | Yes | It is likely that small changes in the assumptions underlying the calculations will reverse these conclusions. It is more reasonable to conclude that, as long as an adequate level of vaccination is reached, it is of less importance who is prioritized. |
| Wang, X | Deaths | E/V | Yes | Prioritisation has limited benefit under low (<50%) uptake. |
| Yu, H | Cases | Y/HC | No | We estimate that the advantage of optimal prioritization strategies over a uniform mass vaccination increases with vaccine supply 2.0 million courses per day (0.14% rollout speed) is reached and decreases as the supply further increases, when the supply is sufficiently large (3.5 million courses; 0.24% rollout speed |
|  | Deaths | E/V |  |  |

Abbreviations – E: elderly, HC: high contacts, SES: socio-economic status, V: vulnerable, VE: vaccine efficacy, Y: younger population

Table S8 - Exceptions to the majority of study conclusions on optimal vaccination priority group to minimise deaths and cases of COVID-19 organised by level of coverage by the end of the modelled vaccination campaign

| Coverage level by end of vx campaign (% pop)​ | Deaths exceptions​ | Cases exceptions​ |
| --- | --- | --- |
| 50-100​ | 4​ | 3​ |
| 30-50​ | ​1 |  |
| 1-30​ | 1​ | ​ |
| N/A​ | 4​ | ​ |
| ​ | 10 | 3 |

Table S9 – Summary of evidence from the systematic literature review review supporting the prioritization of groups in the WHO SAGE vaccine prioritisation roadmap under different supply scenarios during community transmission

| Priority group within the WHO SAGE guideline scenario 1 | No. of studies recommending this group for prioritisation (deaths) | No. of studies recommending this group for prioritisation (cases) |
| --- | --- | --- |
| Essential workers (health or outside health) | 2 | 3 |
| Older adults | 26 | 3 |
| Groups with comorbidities | 1 |  |
| Sociodemographic groups at significantly higher risk of severe disease or death ^[[3]](#footnote-3)^ | 0 | 0 |
| Teachers and school staff | 0 | 0 |
| Pregnant women | 0 | 0 |
| Personnel needed for vaccine production and other high-risk laboratory staff. | 0 | 0 |
| Social/employment groups at elevated risk of acquiring and transmitting infection because they are unable to effectively physically distance (depending on country context) ^[[4]](#footnote-4)^ | 0 | 1 |

1. *Study appears twice

   Also includes: [27] & [28] seniors and middle aged adults, [32] seniors and health workers, [31] seniors at full dose and young adults at quarter dose, [34] young adult and middle aged essential workers, [30] seniors & young and middle aged group in high contact with them, [29] high social contact group & seniors, [26] seniors and comorbidities [↑](#footnote-ref-1)
2. ‘Other’ row: [21] postponing of the second dose, [40] school-going youth, [41] by serology testing, [36] non-vulnerable group

   ^3^ ‘Other’ column**:** [31] allocating a partial dose (by age group), [35] allocation by geographic or group disease burden, [34] age prioritisation only, [41] no serology testing [↑](#footnote-ref-2)
3. Depending on country context, examples may include: disadvantaged or persecuted ethnic, racial, gender, and religious groups and sexual minorities; people living with disabilities; people living in extreme poverty, homeless and those living in informal settlements or urban slums; low-income migrant workers; refugees, internally displaced persons, asylum seekers, populations in conflict settings or those affected by humanitarian emergencies, vulnerable migrants in irregular situations; nomadic populations; and hard-to-reach population groups such as those in rural and remote areas) [↑](#footnote-ref-3)
4. Depending on country context, examples may include: people living or working in detention facilities, incarcerated people, dormitories, informal settlements or urban slums; low-income people in dense urban neighbourhoods; homeless people; military personnel living in tight quarters; and people working in certain occupations such as mining and meat processing) [↑](#footnote-ref-4)
